# Inverted apicobasal polarity in health and disease

**DOI:** 10.1242/jcs.261659

**Published:** 2024-03-11

**Authors:** Nicolas Pasquier, Fanny Jaulin, Florent Peglion

**Affiliations:** ^1^ Collective Invasion Team, Inserm U-1279, Gustave Roussy, Villejuif F-94805, France; ^2^ Cell Adhesion and Cancer lab, University of Turku, FI-20520 Turku, Finland

**Keywords:** Apicobasal polarity, Extracellular matrix sensing, Membrane trafficking, Micropapillary cancer, Monogenic diseases, Embryo implantation

## Abstract

Apicobasal epithelial polarity controls the functional properties of most organs. Thus, there has been extensive research on the molecular intricacies governing the establishment and maintenance of cell polarity. Whereas loss of apicobasal polarity is a well-documented phenomenon associated with multiple diseases, less is known regarding another type of apicobasal polarity alteration – the inversion of polarity. In this Review, we provide a unifying definition of inverted polarity and discuss multiple scenarios in mammalian systems and human health and disease in which apical and basolateral membrane domains are interchanged. This includes mammalian embryo implantation, monogenic diseases and dissemination of cancer cell clusters. For each example, the functional consequences of polarity inversion are assessed, revealing shared outcomes, including modifications in immune surveillance, altered drug sensitivity and changes in adhesions to neighboring cells. Finally, we highlight the molecular alterations associated with inverted apicobasal polarity and provide a molecular framework to connect these changes with the core cell polarity machinery and to explain roles of polarity inversion in health and disease. Based on the current state of the field, failure to respond to extracellular matrix (ECM) cues, increased cellular contractility and membrane trafficking defects are likely to account for most cases of inverted apicobasal polarity.

## Teaser

**Summary:** Review of the molecular alterations associated with inverted apicobasal polarity and their consequences in health and disease.

## Introduction

Cell polarity orchestrates key biological processes such as cell division, cell migration and cell differentiation to ensure tissue morphogenesis and homeostasis, immune defense and wound healing. As such it is a hallmark of all living systems. Cell polarity refers to the asymmetric distribution of molecules and subcellular structures into two opposite poles; this asymmetry typically underlies specialized cellular functions (Nelson, 2003). In human development, the first polarized cells are observed at the 8-to-16-cell blastocyst stage (Gerri et al., 2020a; Nikas et al., 1996). Later, embryos develop to form organs and vasculature composed of a wide range of epithelial and endothelial cell types, all of which share a robust apicobasal polarity. These cells feature an apical pole, which contacts the external milieu or body cavities, and a basal pole, which interacts with the basement membrane and adjacent cells. Through regulation of cell–cell contact positioning, individual cells give rise to the tissue-scale polarity necessary for the barrier and exchange functions of epithelia.

**Fig. 1.**
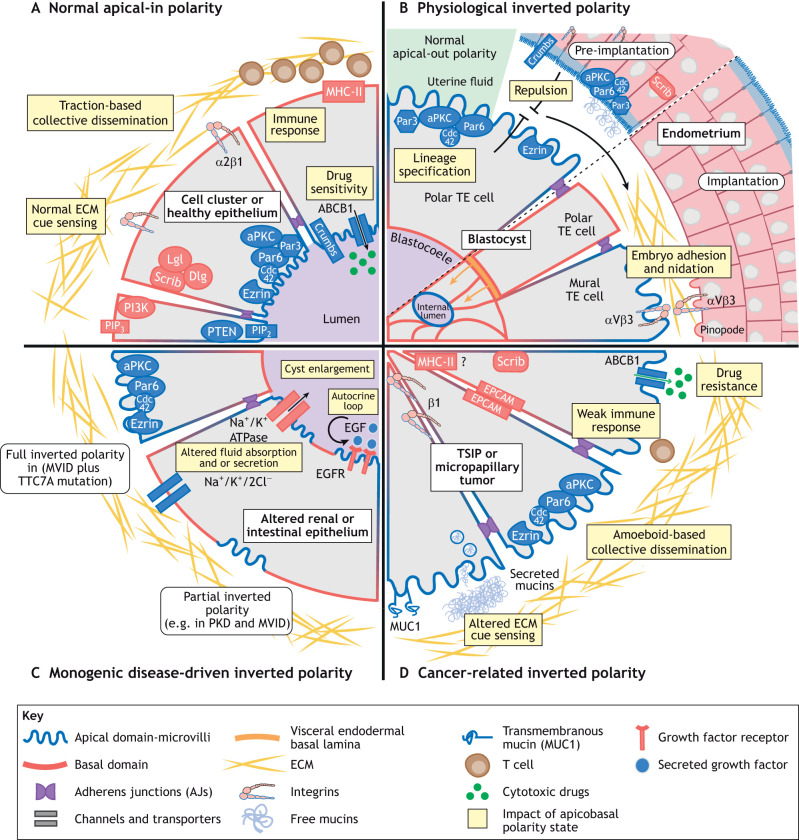
**Hallmarks and consequences of inverted apicobasal polarity in health and disease.** (A) Normal epithelial polarity is regulated by the asymmetric localization of mutually antagonistic complexes. The Par and the Crumbs complexes define the apical pole which is enriched in PIP_2_, PTEN and ezrin (proteins marked in blue). Below AJs, the Scribble complex defines the basolateral domain, which is enriched in PIP_3_ and PI3K (proteins marked in red). ECM sensing through integrins controls the orientation of apicobasal polarity and ensures traction-based collective migration. Basal localization of MHC-II is thought to promote immune clearance of damaged cells by permitting T cell recruitment. Apical localization of the multidrug resistance transporter ABCB1 allows drugs to persist in lumens of epithelia. PIP_3_, phosphatidylinositol 3,4,5-phosphate. (B) The pre-implantation blastula displays apical-out polarity, which prevents adhesion to the uterine wall due to apical–apical repulsion. During the menstrual cycle, to permit successful embryo nidation, apical determinants in the endometrium disappear from the lumen-facing membranes while integrins and pinopodes appear. In parallel, polar throphectoderm (TE) cells invert their polarity in response to emergence of the endodermal basal lamina and mural TE cells express integrins at the periphery of the blastula to promote implantation. (C) MVID enterocytes show partial inverted polarity of microvilli structures. MVID with an additional mutation in *TTC7A* results in fully inverted polarity in these cells. In PKD renal tubules, inverted polarity of ion channels and EGFR contribute to the growth of cysts via altered fluid absorption and secretion. (D) TSIPs arise from micropapillary and mucinous carcinoma. The absence of integrins and presence of mucins at the TSIP periphery prevent cell–ECM interactions resulting in tissue invasion via the collective amoeboid mode of migration. The inverted polarity of ABCB1 enhances cytotoxic drug resistance whereas basolateral localization of MHC-II could limit T cell infiltration and increase immune escape.

The establishment and maintenance of epithelial apicobasal polarity involves dynamic spatiotemporal regulation of a core molecular machinery consisting of protein complexes that are highly conserved across the animal kingdom: the so-called polarity complexes (Fig. 1A) (Buckley and St Johnston, 2022; Peglion and Goehring, 2019; Rodriguez-Boulan and Macara, 2014). In brief, the PAR [Par6, atypical protein kinase C (aPKC) and Cdc42] and CRUMBS [Crumbs, Pals1 and Patj] complexes specify the apical domain, whereas the Scribble complex [Scrib, Lethal giant larvae (LgL) and Discs large (Dlg)] defines the basolateral domain, which is localized below Par3 (also known as PARD3)-enriched cell–cell junctions (Bilder and Perrimon, 2000; Hutterer et al., 2004; Kemphues et al., 1988; Plant et al., 2003; Tanentzapf and Tepass, 2003; Wodarz et al., 1995, 2000). The core polarity machinery, the proteins of which come in multiple forms in mammals, also includes unpolarized cytoplasmic polarity proteins, such as liver kinase B1 (LKB1, also known as STK11; PAR-4 in *Caenorhabditis elegans*) and 14.3.3ζ (encoded by *YWHAZ*; PAR-5 in *C. elegans*) (Baas et al., 2004; Hurd et al., 2003; Winter et al., 2012). Altogether, this hub of protein complexes is able to both read and integrate external polarizing cues to induce self-polarization (Lang and Munro, 2017) and to maintain membrane domain identities by excluding apical complexes from the basolateral plasma membrane and vice versa. For more details on the molecular interaction of the polarity complexes required to build and maintain apicobasal polarity, we refer the reader to several excellent reviews (Buckley and St Johnston, 2022; Martin et al., 2021; Riga et al., 2020; St Johnston, 2018; Stephens et al., 2018). Once activated and in place, the polarity machinery functionally polarizes the entire cell by controlling the spatial organization of the cytoskeleton networks, the asymmetric lipid composition of the plasma membrane and the orientation of membrane traffic, which further reinforces the initial cell asymmetry (Buckley and St Johnston, 2022).

The alteration of apicobasal polarity is associated with numerous pathological conditions, including cancer, autosomal dominant polycystic kidney disease, cystic fibrosis, asthma and viral infections (Tilston-Lunel and Varelas, 2023; Wilson, 1997). Apicobasal polarity perturbations classically include the partial or complete loss of polarity, the extent of which usually correlates with the progression of the disease (Halaoui et al., 2017). In some instances, the polarity alteration might also arise from the inversion of the apical and basolateral domains in cells that maintain an otherwise perfectly polarized state (Box 1). Whereas extensive literature has reviewed how loss of apicobasal polarity contributes to disease progression (Ebnet, 2015), very little is known about the impact of inverted polarity. Orientation of apicobasal polarity relies on the interaction between the core polarity machinery and both the cell–extracellular matrix (ECM) and cell–cell contacts. Changes affecting the biochemical and biomechanical nature of cues from these elements and alteration of the systems that respond to these cues can both lead to abnormal orientation of the apicobasal axis. In this Review, we present a uniform definition of inverted apicobasal polarity based on multiple examples found in physiological and pathological situations, explore its impact on cell and tissue functions and examine the potential origins of inverted polarity from general cell-autonomous or non-cell-autonomous molecular pathways.

**Box 1. Definitions of inverted polarity**

**Normal polarity**

Normal polarity in established epithelial tissues is defined by an apical domain facing the lumen and a basolateral domain contacting the neighboring cells and the basal membrane (bottom left of figure). In cell clusters, polarity is influenced by the extracellular environment. Cell clusters embedded in physiological matrices normally polarize with the apical membranes facing the interior lumen and the outward-facing membrane abutting the ECM (apical-in, top left of figure) (Debnath and Brugge, 2005; Mostov et al., 2003), whereas cell clusters found in extracellular fluid or interstitial spaces normally establish an apical domain on the outward-facing membranes with the basolateral domains facing the interior (apical-out, top center of figure).

**Inverted polarity**

Inverted polarity occurs when some or all components of the apical and basolateral domains are reversed while the overall polarity axis remains intact. This applies to both established epithelial tissues and cell clusters. The term ‘reverse polarity’ is also used by clinicians. Inverted polarity is almost always pathological and the only physiological example is found during mammalian embryo implantation (Sutherland et al., 1993; Whitby et al., 2018).

**Fully inverted polarity**

Full inversion of apicobasal polarity can occur when polarity cues or the response of the cell to the cues are disrupted. In epithelial cells, this means the outward-facing ECM-adjacent membranes are depleted of basolateral proteins and enriched in apical markers such as microvilli and apical polarity proteins (bottom right of figure). This is observed in cancerous aggregates called TSIP (Canet-Jourdan et al., 2022; Zajac et al., 2018), and in established epithelia of individuals with MVID with additional *TTC7A* gene alteration (Michaux et al., 2016). When embedded in physiological matrices, organoids derived from individuals affected by several monogenic diseases also show fully inverted polarity (top right of figure) (Bigorgne et al., 2014; Li et al., 2022; Michaux et al., 2016). Depending on the epithelial cell identity, cavities can sometimes be found within inverted spheroids (not shown here).

**Partially inverted polarity**

In partially inverted polarity, only a subset of apical or basolateral molecules are mispolarized to the opposing domain, whereas the core structural and molecular features of each domain remain intact (bottom center of figure).

**Polarity inversion**

The process by which apicobasal polarity becomes inverted.
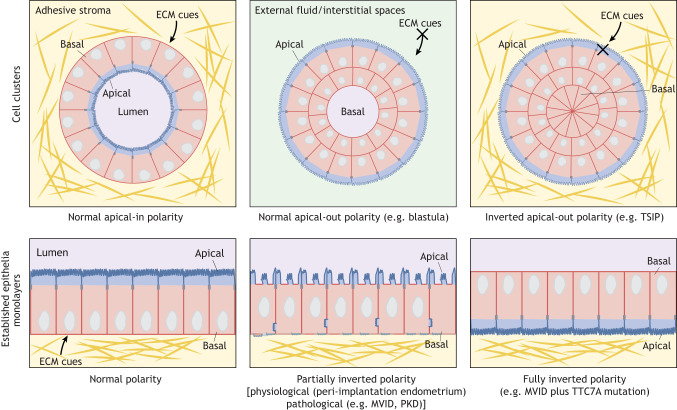

## Inverted polarity in cancer

Apicobasal polarity dictates the polarized functioning of epithelial cells, and loss of apicobasal polarity has long been associated with tumor initiation and invasive progression of carcinoma (cancer deriving from epithelial tissues; Macara and McCaffrey, 2013; Wodarz and Näthke, 2007). Many studies have highlighted that unpolarized epithelia are more prone to carcinoma induction and invasion compared to fully polarized epithelia (reviewed by Lee and Vasioukhin, 2008; Peglion and Etienne-Manneville, 2024). However, histological analysis of tumor specimens has revealed that invasive cancers like colorectal adenocarcinomas display clearly differentiated morphologies with intact polarized epithelial structures that delineate internal luminal cavities within the neoplastic glands (Libanje et al., 2019). Whether the presence of inverted apicobasal polarity in such structures bears consequences for carcinoma pathophysiology is an increasingly explored hypothesis (Peglion and Etienne-Manneville, 2024).

Neoplastic epithelial glands in a subset of highly invasive cancers called micropapillary carcinoma exhibit fully inverted apicobasal polarity (Verras et al., 2022). This type of cancer is diagnosed upon detection of an apical-out polarity pattern. In these tumors, the apical transmembrane glycoprotein MUC1 [also known as epithelial membrane antigen (EMA)] is found at the tumor periphery, and the basolateral protein epithelial cell adhesion molecule (EPCAM) is found on the inward-facing membranes (Fig. 1D; Table 1). In addition to being well-described in breast and lung carcinomas (Adams et al., 2004; Hirakawa et al., 2022; Luna-Moré et al., 1994; Nassar et al., 2004; Siriaunkgul and Tavassoli, 1993), these inverted structures are also found in colorectal (Verdú et al., 2011), cervical (Stewart et al., 2018) and thyroid carcinomas (Asioli et al., 2013). Furthermore, epithelial structures with inversely polarized nuclei are typically seen in the breast solid papillary carcinoma with reverse polarity (SPCRP) tumor subtype (Chiang et al., 2016). Apically localized nuclei in single-layered eosinophilic cells is a feature also used to diagnose papillary renal neoplasm with reverse polarity (PRNRP), a type of kidney cancer with typically good prognoses (Al-Obaidy et al., 2019; Al-Obaidy et al., 2020; Kim et al., 2020).

**Table 1.**
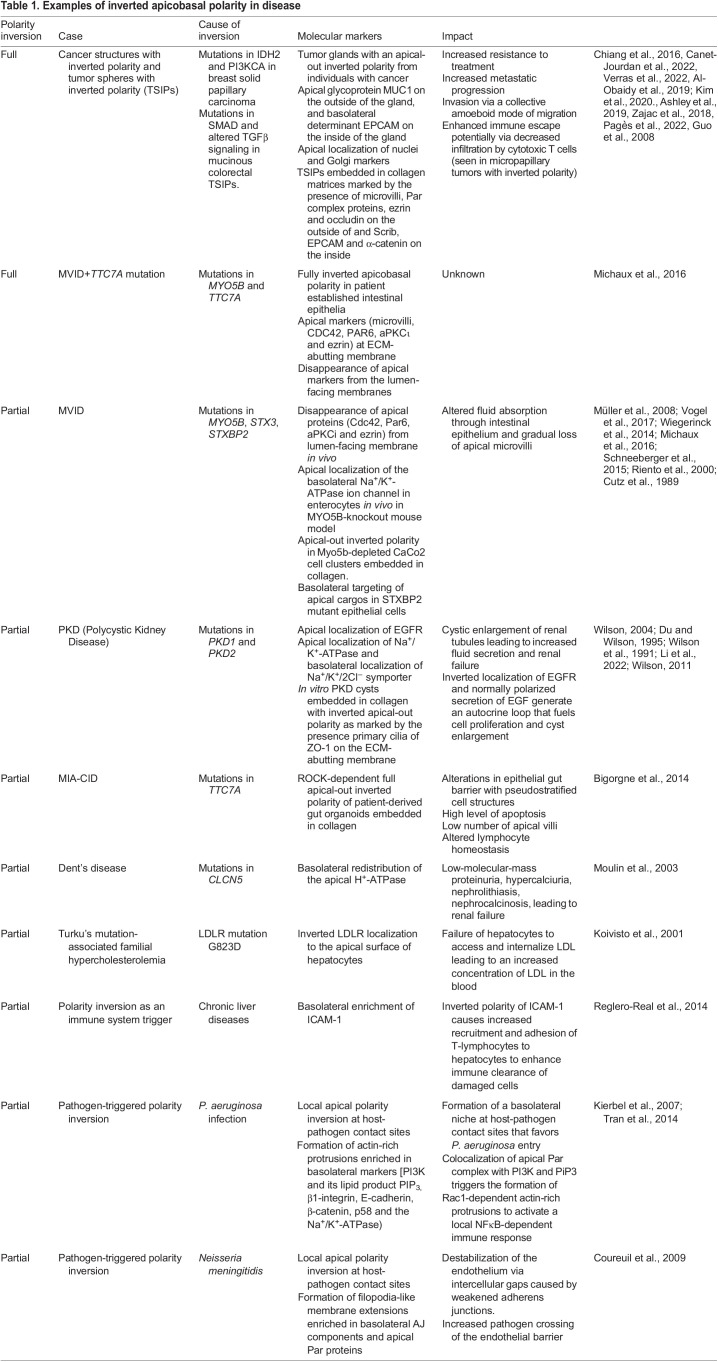
Examples of inverted apicobasal polarity in disease

In addition, cancer clusters with apical-out polarity are often found in non-adhesive tissue interfaces (Box 1). Examples include clusters in suspension in peritoneal or pleural effusions (Ritch and Telleria, 2022; Zajac et al., 2018), in the lumen of lymphatic vessels, in lymph nodes (Mohammed et al., 2019; de Boer et al., 2010) and in pools of mucins (Sun et al., 2020). These clusters were originally called tumor spheres with inverted polarity (TSIP), even though apical-out polarity is typically a normal feature of cell clusters in suspension. For some individuals with cancer, however, clusters retrieved from non-adhesive substrates maintain their apical-out polarity even when placed in 3D physiological matrices, suggesting a genuine inverted apicobasal polarity phenotype (Canet-Jourdan et al., 2022; Okuyama et al., 2016; Onuma et al., 2021; Zajac et al., 2018).

The exact role played by apicobasal polarity inversion in cancer initiation and progression is still poorly understood. Functional studies have revealed that variants in *IDH2* and *PIK3CA* oncogenes in the breast SPCRP tumor subtype are sufficient to elicit the inverted polarity phenotype in healthy breast organoids (Chiang et al., 2016). These data suggest apicobasal polarity inversion could directly contribute to cancer initiation and progression.

### Roles in cancer invasion

Polarity-inverted micropapillary carcinomas are highly infiltrative cancers with a high incidence of lymph node metastasis (Kuroda et al., 2004). More recently, our laboratory has identified TSIPs as the malignant intermediates in the metastatic spread of micropapillary and mucinous colorectal cancers (Zajac et al., 2018). That study demonstrated that TSIPs are migratory neoplastic structures on their way to colonizing secondary organs. Identified in the peritoneal fluids of individuals with highly metastatic disease, TSIPs are defined by having an apical-out topology along the entire course of their metastatic spread in fluids and tissues, as seen during peritoneal invasion *ex vivo* and in a mouse colorectal liver metastasis model (Zajac et al., 2018). They are unable to sense and strongly adhere to the ECM due to the absence of integrins on their outward-facing membranes, and TSIPs do not generate actin-based protrusions. This raises the question of how TSIPs collectively migrate and disseminate if they cannot perform traction-based migration (Fig. 1A,D). TSIPs were found to propagate by self-generating smaller cell clusters that bud out from the mother TSIP (Zajac et al., 2018). Most interestingly, a recent study suggests that TSIPs can actively invade the matrix using a collective amoeboid mode of migration (Pagès et al., 2022). Using non-adhesive microchannels, Pagès et al. revealed that the entire TSIP propels itself in a confined environment in a similar fashion to single immune cells and amoeba. Collective amoeboid migration relies both on friction forces generated by the fluctuating deformation (‘jiggling’) of the peripheral cells and the polarized enrichment of a contractile supracellular cortical actomyosin cap spanning multiple cells at the rear of the cluster (Pagès et al., 2022). The ability of TSIPs, and more globally of tumor clusters, which both lack the specific adhesion receptors required to interact with the surrounding ECM, to keep migrating in this atypical fashion likely fuels tumor invasion. Even partial apicobasal polarity inversion following β1-integrin depletion is sufficient to trigger long-term invasion of Madin–Darby canine kidney (MDCK) spheroids into Matrigel^®^ in a collective amoeboid fashion (Bryant et al., 2014), reinforcing that the absence of ECM sensing does not prevent cell clusters from invading tissues. Whether the hypothesis that pro-metastatic ‘all-terrain’ collective amoeboid migration holds true *in vivo* remains to be addressed.

### Cancer growth and treatment resistance

Inverted apicobasal polarity also affects tumor growth and treatment resistance. Primary colorectal cancer organoids with an inverted apical-out phenotype better survive chemotherapeutic treatment compared to normally polarized apical-in organoids (Canet-Jourdan et al., 2022). This could be due to a reduced proliferation rate in TSIPs compared to apical-in organoids, making them less susceptible to anti-mitotic drugs (Canet-Jourdan et al., 2022). Another explanation could be that TSIPs are better protected structurally against cytotoxic drugs. Normally polarized apical-in cancer clusters display apically located multidrug resistance transporters, such as the efflux transporter ATP binding cassette subfamily B member 1 (ABCB1), on the lumen-facing membranes. ABCB1 drug substrates thus accumulate in the cluster lumen, which might prolong their cytotoxic effects (Ashley et al., 2019) (Fig. 1A). In TSIPs, ABCB1 is expressed on the outward-facing membranes, and drug substrates are therefore excluded more easily (Ashley et al., 2019) (Fig. 1D).

### Cancer immune escape

Epithelial cells act as non-professional antigen-presenting cells and participate in immune surveillance and response (Wosen et al., 2018). Antigen-presenting membrane receptors, such as the major histocompatibility complex class II (MHC-II) receptors, display a polarized location in intestinal and lung epithelial cells. Multiple *in vitro* and *in vivo* human studies have revealed that MHC-II proteins such as HLA-DR and HLA-DM are restricted to the basolateral surfaces of intestinal epithelial cells (Hirata et al., 1986; Sarles et al., 1987; Wosen et al., 2019). Their polarized localization enables activation, modulation or maintenance of CD4+ T cells (Hershberg et al., 1998), whose role in anti-tumor immunity is increasingly being recognized (Speiser et al., 2023). Alteration of MHC-II signaling plays a central role in immune evasion during cancer development and is regarded to be a causal factor for immunotherapy failure (Axelrod et al., 2019). Owing to their inverted apical-out, basal-in polarized state, mucinous and micropapillary carcinomas are expected to be depleted of MHC-II molecules on their outward-facing membranes, thus reducing accessibility for resident T-cells. Whether the altered presentation of MHC-II proteins contributes to immune escape in mucinous and micropapillary carcinoma remains a hypothesis worth addressing. Interestingly, invasive micropapillary breast carcinomas are less infiltrated by cytotoxic T cells compared to normally polarized medullary breast carcinomas (Guo et al., 2008) (Fig. 1D).

Toll-like receptors (TLRs) are another family of antigen recognition receptors involved in triggering immune responses, mostly during pathogen infection. TLRs also impact cancer progression, either positively or negatively depending on the identity of the TLR. For example, TLR-3 stimulation triggers apoptosis and directly kills human cancer cells (Salaun et al., 2006). In human intestinal epithelial cells, TLR-3 localization is restricted to the basolateral domain, enabling an asymmetric immune response in which apical commensal bacteria are tolerated while crossing of the epithelium and basolateral intrusion of pathogens is actively guarded against (Stanifer et al., 2020). A tempting untested hypothesis is that the absence of TLR-3 on the outward-facing apical membrane of TSIPs in intestinal tumors could alter the anti-tumoral activity of TLR-3 and enhance tumor immune escape.

Apicobasal polarity inversion is thus a multifaceted feature of invasive cancers. Although to what extent inverted polarity plays a role in tumor initiation remains unknown, accumulating evidence suggests it is involved in cancer progression by promoting all-terrain migration, treatment resistance and immune escape. Further work is needed to confirm untested hypotheses and reveal the molecular processes explaining how inverted polarity drives aggressive cancers.

## Inverted polarity in genetic diseases

Inverted polarity structures also occur in epithelial tissues of many organs impacted by monogenic diseases. One of the clearest examples is the microvillus inclusion disease (MVID), a genetic disorder affecting intestinal absorption, which often causes fatal watery diarrhea. MVID is characterized by a gradual loss of apical microvilli and the formation of microvilli inclusions inside enterocytes (Cutz et al., 1989). In 90% of cases, MVID is caused by gene variants of Myosin Vb (*MYO5B*), with variants of syntaxin-3 (*STX3*) and its binding partner syntaxin binding protein 2 (*STXBP2*) are responsible for the remaining 10% of cases (Müller et al., 2008; Vogel et al., 2017; Wiegerinck et al., 2014). Depletion of Myo5B in an MVID mouse model causes a partially inverted polarity phenotype with total loss of apical microvilli, an inverted apical localization of the Na^+^-K^+^ pump (Na^+^/K^+^-ATPase) and basolateral presence of E-cadherin in enterocytes (Schneeberger et al., 2015) (Fig. 1C, Table 1). Interestingly, two individuals with MVID that have an additional mutation in the tetratricopeptide repeat domain 7A (*TTC7A*) gene of unknown function display complete inversion of apical polarity determinants, such as microvilli, Cdc42, Par6, aPKCι and ezrin in the intestinal epithelium (Michaux et al., 2016) (Fig. 1C, Table 1).

A rare bowel obstruction disease called multiple intestinal atresia associated with combined immunodeficiency (MIA-CID) is linked to variants in the same *TTC7A* gene. Studies on intestinal organoids derived from cells from individuals with MIA-CID have revealed that TTC7A deficiency causes a Rho-associated protein kinase (ROCK; herein referring to both ROCK1 and ROCK2)-dependent full inversion of apicobasal polarity (Bigorgne et al., 2014). Whether the inversion of polarity is involved in the defects in cell growth, intestinal epithelial differentiation and immune cell homeostasis remains to be investigated.

Partially inverted apicobasal polarity is also observed in the ciliated kidney epithelium of individuals with polycystic kidney disease (PKD). In PKD, normal renal tubules are replaced by fluid-filled cysts which dissociate from the nephron, expand and proliferate, leading to end-stage renal disease (Menezes and Germino, 2019; Simons and Walz, 2006). PKD is mainly caused by autosomal dominant (AD) mutations in one of the two genes, *PKD1* and *PKD2*, encoding for ubiquitously expressed polycystin-1 (PC1), a transmembrane protein, and polycystin-2 (PC2), an ion channel (Wilson, 2004). How these mutations initiate the polycystic phenotype remains unresolved (Ong, 2017). Nevertheless, a consensus exists on the crucial role played by the inversion of apicobasal polarity in tubular renal epithelial cells. Many basolateral proteins, such as the epidermal growth factor receptors (EGFRs), are enriched at the apical luminal pole in epithelial cells that line PKD cysts (Wilson, 2011). The presence of EGFR together with its ligand EGF, which is normally secreted from the apical luminal pole, generates an autocrine loop that fuels cell proliferation and cyst enlargement (Du and Wilson, 1995). In PKD cysts, Na^+^/K^+^-ATPase and the Na^+^/K^+^/2Cl^−^ symporter switch their polarity, which is thought to lead to increased fluid secretion into the cyst lumen and enlargement of the cysts (Wilson et al., 1991). Recently, analysis of results from a PKD-kidney-on-chip model, in which cysts that detached from peripheral tubular epithelium embedded in collagen present a fully inverted apical-out phenotype, has suggested an alternative mechanism (Li et al., 2022). The authors propose that the inverted polarity fuels absorption of glucose and fluids from the cyst periphery, leading to increased internal pressure, epithelium stretching and cyst expansion (Fig. 1C). Evidence suggests that a partial apicobasal polarity inversion involving a restricted number of polarized proteins could also lead to pathological conditions in several other monogenic disease (see Table 1).

## Inverted polarity in immune response and pathogen defense

Specific instances of inversion of apicobasal cell polarity are required in mammalian organisms to regulate epithelial homeostasis and respond to pathogen infection. Next, we will discuss several examples of inverted polarity found to be involved in inflammation and the immune responses to pathogens.

The partial polarity inversion of damaged and dysfunctional epithelia can act as a signal for unhealthy cells to be cleared by the immune system. Healthy hepatocytes segregate intracellular adhesion molecule 1 (ICAM-1) at the apical pole, which faces the bile caniculi lumen (Reglero-Real et al., 2014). Individuals with chronic liver disease often display inverted ICAM-1 localization in basally located microvilli-like structures, a phenotype that has been reproduced experimentally by treatment with pro-inflammatory tumor necrosis factor α (TNFα; also known as TNF). In this study, the inverted polarity of ICAM-1 increased the recruitment and adhesion of T-lymphocytes to hepatocytes (Reglero-Real et al., 2014). Here, the apicobasal polarity inversion is thus harnessed to favor adhesion of leukocytes and promote faster clearance of the damaged cells displaying inverted polarity.

Changes in apicobasal intestinal epithelial and endothelial polarity are also observed during early stages of pathogen infection (Tapia et al., 2017). In some instances, the plasma membrane that makes direct contact with bacterial aggregates loses its apical identity and expresses basolateral markers. The most striking example of such polarity inversion has been observed during often-lethal infection by *Pseudomonas aeruginosa* bacteria. Using polarized monolayers of MDCK cells, Kierbel et al. found *de novo* formation of cell protrusions around bacterial clusters at the apical pole of the cells. These protrusions are devoid of the apical sialomucin gp135 (also known as podocalyxin) and enriched in basolateral determinants, including phosphoinositide 3-kinase (PI3K) and its lipid product phosphatidylinositol (3,4,5)-trisphosphate (PIP_3_), β1-integrin, E-cadherin, β-catenin and p58 (also known as the Na^+^/K^+^ ATPase transporting subunit β, ATP1B1) Na^+^/K^+^-ATPase (Kierbel et al., 2007). *P. aeruginosa* exploits the ability of PIP_3_ to convert apical membranes to a basolateral identity in order to create a more favorable nesting microenvironment by activating PI3K (Gassama-Diagne et al., 2006; Kierbel et al., 2005). Intriguingly, the Par3–Par6–aPKC apical polarity complex remains present in the actin-rich protrusions surrounding bacterial aggregates. The presence of this complex is thought to locally activate Rac1, which, together with PI3K, induces NF-κB activity and downstream immune response signaling (Tran et al., 2014). Although this spatially restricted inversion of polarity at the host–pathogen contact site enables entry of *P*. *aeruginosa* into host cells, it could also thus act as a ‘danger signal’ for the host organism to hasten detection of the infected epithelial cell.

Local inversions of polarity are also sometimes used by pathogens to alter endothelial barrier integrity and fuel aggressive infection. Aggregates of *Neisseria meningitidis* bacteria, the causative agent of cerebrospinal meningitis, induce plasma membrane remodeling at host–pathogen contact sites. Newly formed filopodia-like membrane extensions are enriched in both basolateral adherens junction (AJ) components and apical Par proteins (Coureuil et al., 2009). Here, unlike what occurs upon infection with *P. aeruginosa*, the presence of ectopic basolateral cell–cell adhesions at the apical pole destabilizes the endothelium, opens intercellular gaps and promotes crossing of the endothelial barrier by *N*. *meningitidis* (Coureuil et al., 2009). Thus, inverted polarity plays a role in tissue homeostasis and integrity, but this feature can also be maliciously hijacked to initiate and/or fuel disease progression.

## Inverted polarity in development

Outside the context of disease, inversion of apicobasal cell polarity is a biological process used at key moments of mammalian development. Early embryonic development and nidation (implantation of the embryo into the uterine wall) are some of the clearest examples of physiologically relevant inversion of apicobasal polarity (see Box 2 and Box 3). From the eight-cell stage in mice and humans, the blastomeres of the embryo start to compact and develop an apical-out polarity marked by the asymmetrical localization of microvilli, actin, actin-binding proteins and the Par3–Par6–aPKC complex on the outward-facing membranes of the blastocyst (Ducibella et al., 1977; Lehtonen and Badley, 1980; Nikas et al., 1996; Plusa et al., 2005; Reeve and Ziomek, 1981; Vinot et al., 2005), and the localization of basolateral proteins Scrib and Llg1 at cell–cell contacts (Hirate et al., 2013). The polarized blastomeres (or polar cells) retain this apical pole during successive divisions and later differentiate to form the trophectoderm (TE), the first apicobasally polarized sheet of cells, which will become the placenta (Box 2) (Johnson and Ziomek, 1981; Suwińska et al., 2008). During the pre-implantation period, the embryo is in contact with the external milieu and the presence of an apical domain on its outer surface reflects a normal apical-out polarity state (see Box 1). During implantation, the apical-out polarized TE cells need to adhere to the luminal epithelial cells of the endometrium for successful nidation of the embryo (Thie et al., 1996). As implantation proceeds, both the embryo and the endometrium modify their polarity to prevent polar repulsion caused by apical–apical sensing (Fig. 1B). On one side of the embryo, a pool of polar TE cells inverts their apicobasal polarity at a tissue-scale level. This transition is caused by rapid cell proliferation and expansion of the visceral endodermal basal lamina, whose polarizing cues trigger the generation of an internal apical domain within the embryo and the disappearance of apical markers from the outward-facing TE cell membranes (Ozguldez et al., 2023) (Fig. 1B). On the opposite side of the embryo, the mural trophoblast cells directly contacting the uterine wall maintain their apical domain on the outward-facing surface but express and display an inverted localization of αVβ3 integrins, which engage with laminin ligands upregulated in the uterine environment, to promote trophoblast attachment (Sutherland et al., 1993) (Fig. 1B). In parallel, the receptivity of the endometrium is controlled by hormone-dependent changes in apicobasal polarity of the luminal epithelium (Whitby et al., 2020). Apical enrichment of Par proteins, Crumbs and mucins, and basolateral enrichment of Scrib progressively disappear during the menstrual cycle (Whitby et al., 2018). Furthermore, instead of forming microvilli, the apical membrane of endometrial cells reorganizes into smooth bulbous projections called pinopodes, the abundance of which peaks at the end of the secretory phase, when the endometrium is receptive to implantation (Quinn et al., 2020). Concomitantly, together with the apical secretion of the αVβ3 integrin ligand osteopontin, which is crucial for blastocyst implantation (Illera et al., 2000), basolateral adhesive molecules, such as αVβ3 integrin itself, switch their polarized localization and become enriched in the lumen-facing apical domain (Aplin et al., 1996; Lessey, 2002) (Fig. 1B). Here, hormone-dependent partial inversion of polarity is used to transiently adapt the adhesive needs of both the uterine wall and the embryo for successful nidation and pregnancy. Taken together, the examples above can be seen as physiologically relevant instances of polarity inversion at play during successful development.

**Box 2. Apicobasal polarity in the mammalian embryo**

Specification of polar and apolar cells within the embryo is key to defining and maintaining the overall polarity axis. Cell localization depends on differences in cortical tension, cell signaling and modes of cell division. During embryo compaction, cells exhibiting higher cortical tension are pushed inwards and will not polarize, whereas cells with lower cortical tension develop the apical domain on the outside of the embryo (Maître et al., 2016; Samarage et al., 2015). Asymmetric division leading to differential inheritance of the apical domain increases the pool of apolar cells and directs their positioning inside the embryo, while maintaining polar cells on the outside (Maître et al., 2016). Symmetric division is also crucial to extending the apical domain at the embryo periphery as the blastocyst grows (Niwayama et al., 2019). Emergence of an aPKC-rich apical domain facing the external milieu is suggested to be key to controlling early lineage specification by orchestrating cell positioning, cell contractility and the orientation of cell division (Gerri et al., 2020b; Zhang and Hiiragi, 2018). The crosstalk between the Par complex and the Hippo pathway is crucial for coupling the structural and functional polarization of the embryo. The core Hippo pathway is a kinase cascade leading to the cytoplasmic sequestration of the YAP/TAZ transcription factors to control cell proliferation, organ size and pluripotency of embryonic stem cells (Badouel and McNeill, 2011). In polar cells, Par6b and aPKC suppress Hippo signaling thereby enhancing the expression of the YAP downstream target gene *Cdx2,* a marker of trophectoderm (TE) cells, which acts as an inhibitor of Nanog and Oct4 (also known as POU5F1), two stemness markers. This molecular cascade explains how cell polarity restricts the activation of stemness markers in the apolar cells of the inner cell mass (ICM) (Anani et al., 2014; Hirate et al., 2013; Mo et al., 2014). Further into blastocyst development, some internal ICM cells mature, start expressing polarity genes, self-organize on the periphery of the ICM and later form the primitive endoderm. The remaining non-polarized cells become the epiblast or embryo per se (Zhu and Zernicka-Goetz, 2020).

**Box 3. The intriguing case of the first lumen generated during mammalian embryonic development**

Early embryonic development includes an extreme case of polarity inversion whereby both apical and basolateral domains face extracellular fluids. At the blastocyst stage, trophectoderm (TE) cells envelop a fluid-filled lumen called the blastocoele cavity which bathes the apolar inner cell mass (ICM) cells. At the onset of blastocoel formation, fluids and osmolytes are transported and secreted asymmetrically to the basolateral compartment of internal cells as a result of basolateral enrichment of fluid pumps like the Na^+^/K^+^-ATPase (Hirata et al., 1986; Schliffka and Maître, 2019). The increasing fluid pressure breaks basolateral cell–cell bonds, creating small cavities which coalesce into a single blastocoele lumen at the TE–ICM interface. This process is controlled by anisotropies in cell contractility and cell adhesion. Because the ICM is more contractile than the TE, extracellular fluid is thought to be directed towards the softer tissue to generate the single blastocoele cavity (Dumortier et al., 2019). As such, the first lumen-facing membrane during development has a basolateral identity, revealing an interesting case of inverted apicobasal polarity in which both the apical and the basolateral domains face a fluid external milieu.

## Molecular determinants of apicobasal polarity inversion

**Fig. 2.**
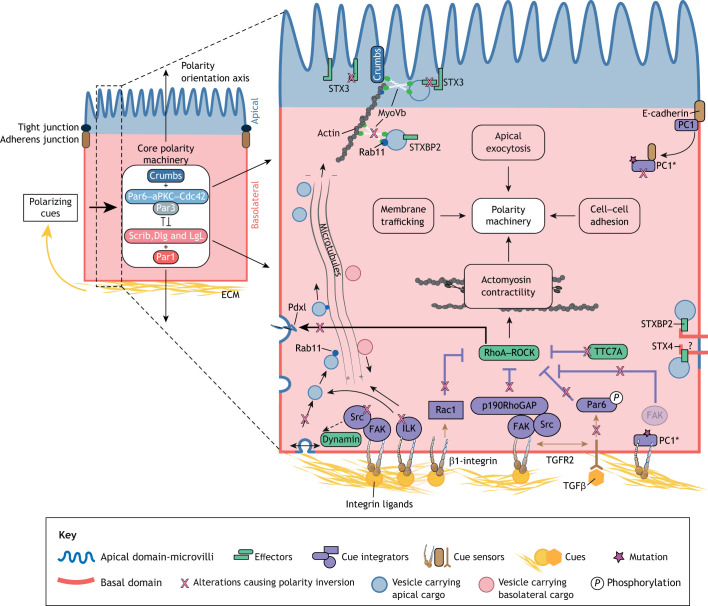
**Molecular pathways controlling apicobasal polarity inversion.** Polarizing cue sensing and integration by epithelial cells controls how the apicobasal polarity machinery establish and maintain the appropriate polarity state. The enlarged view of the cell on the right shows how basolateral integrins and growth factor receptors sense ECM adhesive cues and growth factors (orange). Multiple cue-integration pathways (purple) trigger local activation of effectors (green), which control the orientation of the polarity machinery. Integrin engagement activates FAK and Src kinases, which downregulate RhoA activity to reduce actomyosin contractility. Decreased RhoA activity directly promotes the endocytosis and Rab11–Cdc42-dependent transcytosis of apical determinants, such as Pdxl (blue vesicles). Integrin engagement also activates ILK, which repolarizes the microtubule network to favor endocytosis and trafficking of apical determinants. TGFβ signaling via TGFR2 downregulates RhoA activity through phosphorylation of Par6. Impairments in these pathways (pink Xs) cause various degrees of apicobasal polarity inversion. In MVID, mutations in STX3, STXBP2 and Myo5B are thought to cause inverted trafficking of apical cargo towards the basolateral domain. The binding of apical vesicles to STX4, a close homolog of STX3 located basolaterally, could contribute to inverted polarity associated with STX3 mutations. In PKD, PC1 mutations prevent FAK from associating with activated integrins in focal adhesions. Alternatively, mutant PC1 is known to sequester E-cadherin in the cytoplasm, preventing the sensing of polarizing cues from adherens junctions.

The molecular mechanisms underlying establishment and maintenance of apicobasal polarity are now well documented (Buckley and St Johnston, 2022). In contrast, the regulation of the orientation of the polarity axis is less well understood. Lumen-forming epithelia and cell clusters generally polarize in a basal-to-apical fashion by integrating dominant polarizing cues from the ECM (Martin-Belmonte and Mostov, 2008) (Fig. 2). We will next explore the molecular drivers of apicobasal polarity inversion in the aforementioned pathological conditions to unveil the critical pathways controlling epithelial polarity orientation.

### Failure to read and integrate polarizing ECM cues drives basal-to-apical polarity inversion

Seminal work from the Nelson laboratory has revealed the importance of the ECM in dictating the orientation of the apicobasal polarity axis. Aggregates of MDCK cells in suspension switch from an apical-out to an apical-in polarity phenotype upon inclusion in collagen gels, providing an excellent model to study the spatiotemporal events responsible for normal emergence of apicobasal polarity (Wang et al., 1990a,b). These studies have implicated β1-integrin signaling in translating ECM cues via activation of Rac1 GTPase (Ojakian and Schwimmer, 1994; Yu et al., 2005). Follow-up studies highlight the importance of downregulating the activity of RhoA and ROCK, and hence actomyosin contractility, upon integrin engagement to successfully drive cell cluster polarization, independently of Rac1 activity (Ferrari et al., 2008). In brief, ECM ligands sensed by β1-integrin activate focal adhesion kinase (FAK; also known as PTK2) at focal adhesions, which recruits p190RhoGAP (also known as p190A or ARHGAP35) to locally inhibit RhoA at the ECM-abutting membrane (Bryant et al., 2014). Decreased activity of RhoA and ROCK destabilizes the anti-adhesive apical sialomucin gp135 (also known as podocalyxin; Pdxl), which, along with the Par6–aPKC–Par3 complex, is internalized and transcytosed in a Rab11–Cdc42–exocyst complex-dependent fashion to the opposite membrane to initiate formation of a lumen (Bryant et al., 2010, 2014; Martin-Belmonte et al., 2007) (Fig. 2). Delivery of the apical membrane components to the newly formed lumen-abutting domain also relies on Rab35, either via its capacity to tether gp135-positive vesicles (Klinkert et al., 2016) or via its regulation of the small GTPase Arf6 (Mrozowska and Fukuda, 2016). Intestinal organoids derived from individuals with MIA-CID with inhibitory variants in *TTC7A* show an inverted apical-out polarity phenotype when embedded in 3D matrices (Bigorgne et al., 2014). Interestingly, inhibiting ROCK-dependent actomyosin contractility is sufficient to restore normal apical-in polarity, reinforcing the key role of RhoA–ROCK activity in controlling apicobasal polarity orientation (Bigorgne et al., 2014). Alternatively, to achieve the apical-out to apical-in polarity inversion upon 3D matrix inclusion, clusters of mammary gland epithelial cells require β1-integrin-dependent activation of the integrin-linked kinase (ILK) and microtubule polarity inversion (Akhtar and Streuli, 2013). Activation of ILK stabilizes microtubule plus-ends at the outward-facing surface, which enhances endocytosis and removal of apical determinants from the cluster periphery. Furthermore, by re-orienting microtubule minus-ends away from the outward-facing surface, the β1-integrin–ILK–microtubule network promotes repositioning of the Golgi and membrane trafficking of apical components from the periphery to the newly forming lumen-facing membrane (Akhtar and Streuli, 2013) (Fig. 2). For more details on the role played by microtubules and associated molecular motors in controlling polarity inversion, we refer readers to the excellent review by Kreitzer and Myat (2018). The diversity of signaling pathways that are involved in polarity inversion downstream of β1-integrin likely reflects the different experimental settings, including the nature of ECM molecules (collagen versus Matrigel) and differing epithelial cell types (kidney versus mammary) used in the experiments.

The study of organoids derived from individuals with colorectal cancer (CRC) has unveiled several other key molecular alterations leading to polarity inversion. Among them, the lack of active canonical and non-canonical transforming growth factor β (TGFβ) signaling is key to explaining the inverted apical-out polarity orientation of 3D matrix-embedded CRC clusters both *in vitro* and in host tissue following mouse xenografting (Canet-Jourdan et al., 2022; Zajac et al., 2018). This likely results from several downstream mechanisms including an altered positive-feedback loop between TGFβ and integrin expression levels (Munger and Sheppard, 2011). Downstream of integrin engagement, actomyosin contractility plays a crucial role in controlling the apicobasal polarity state of CRC clusters in 3D matrices. Downregulation of RhoA activity is sufficient to restore normal apical-in polarity in CRC clusters in 3D matrices, whereas increased contractility in initially normal apical-in polarized clusters drives polarity inversion (Canet-Jourdan et al., 2022; Onuma et al., 2021; Zajac et al., 2018). An interesting candidate which might bridge the TGFβ signaling module with inhibition of the RhoA–ROCK effector module is the polarity protein Par6. It was previously reported in mammary gland epithelial cells that upon binding with its ligand, the TGFβ receptor complex phosphorylates Par6, which increases the interaction of Par6 with the E3 ubiquitin ligase Smurf1 and causes the local degradation of RhoA (Ozdamar et al., 2005). Zajac et al. found that Par6 depletion partially blocks TGFβ-treated CRC clusters from establishing a normal apical-in polarity in collagen matrices (Zajac et al., 2018), suggesting that a similar molecular cascade could be at play to control apicobasal polarity orientation in cell clusters (Fig. 2). Alterations in other growth-factor-dependent signaling pathways might also be involved, as EGF and insulin growth factor-1 (IGF-1) have been shown to control lumen formation upon inclusion of CRC organoids in 3D matrices (Ashley et al., 2019). Finally, pharmacological inhibition of FAK and microtubule dynamics do not significantly prevent lumen formation and apical-in polarity of CRC clusters embedded in collagen, in contrast to what is seen for MDCK cells and mammary acini, suggesting additional pathways are involved in polarity inversion in colorectal cancer (Okuyama et al., 2016). Treating CRC clusters with the Src inhibitor Dasatinib or inactivating dynamin through addition of Dynasore and MitMAB strongly block the restoration of an apical-in polarity state, suggesting that Src kinase and dynamin play a role in controlling polarity inversion downstream of β1-integrin engagement with ECM molecules (Okuyama et al., 2016) (Fig. 2). Src kinase can directly activate the GTPase activity of dynamin2 (Dyn2; also known as DNM2) (Weller et al., 2010). In addition to its role in promoting cell membrane endocytosis, Dyn2 regulates Rac1 activity by stabilizing its guanine nucleotide exchange factor (GEF) Vav1 (Razidlo et al., 2013). Whether Src and dynamin act together downstream of β1-integrin to enhance Rac-1 mediated inversion of polarity remains to be tested.

Apical-out inverted polarity in tumor clusters could also be fueled by the altered tumoral microenvironment. For example, colorectal TSIPs secrete mucus at their periphery, which isolates the tumor cluster from polarizing ECM cues, further stabilizing the apical-out polarity (N.P., Jacques Mathieu, Johanna Ivaska, F.J., unpublished). In other cases, increased stiffness of the stroma (the noncancerous tissue which surrounds and supports a tumor), which is often associated with carcinoma progression, could foster TSIP formation. Augmenting matrix stiffness triggers the partial inversion of apicobasal polarity in mammary epithelial cell clusters via increased integrin-dependent focal adhesion signaling, which enhances growth factor-dependent extracellular signal-regulated kinases 1 and 2 (ERK1/2) activation and downstream RhoA activity (Paszek et al., 2005). Decreasing actomyosin contractility rescues the polarity defects in malignant and non-malignant mammary epithelial cell clusters caused by high matrix stiffness (Paszek et al., 2005). These two examples highlight the role played by the tumor environment in potentiating the cell-autonomous molecular alterations that cause the inverted polarity phenotype.

Altogether, these studies on matrix-embedded cell clusters have revealed key molecular cascades at play during apicobasal polarity inversion. Alterations in cue-sensing via integrins and the TGFβ receptor, in integration of ECM cues via Src, FAK, ILK, Par6 or Rac, and in effector modules, such as actomyosin contractility and membrane trafficking, might all contribute to failure to establish and maintain a normal apicobasal polarity orientation (Fig. 2). Whether these mechanisms also control the orientation of apicobasal polarity in established epithelia requires further investigation. Early studies in partially inverted renal epithelia in individuals with PKD suggest that ECM cue-sensing via integrins and cue integration via FAK could potentially be involved. In normal renal tubules, the polycystin protein PC1 (also known as PKD1) interacts with α2β1 integrins at focal adhesions (Wilson et al., 1999). In cells individuals with PKD with *PC1* gene variants, FAK is absent from the focal adhesion macromolecular complex, suggesting transmission of polarizing ECM cues is flawed due to dysfunctional focal adhesions (Wilson, 2011).

### Alteration of apical membrane trafficking primes epithelia for partial apical-to-basal polarity inversion

In a vast majority of cases, the epithelia affected by partial or full apicobasal polarity inversion suffer from defects in membrane trafficking and vesicle recycling. For example, *P. aeruginosa* infection triggers PI3K-dependent transcytosis of basolateral markers, such as E-cadherin, integrin and PIP_3_, towards the lumen-facing pole, as well as displacement of the apical marker gp153 (Kierbel et al., 2007; Tran et al., 2014). The exact molecular mechanisms responsible for this inverted targeting of apical and basal membrane determinants remain poorly understood. Next, we discuss findings that have begun to point toward these elusive mechanisms.

One scenario involves defective apical endosome recycling (Schneeberger et al., 2018). MYO5B, variants in which cause MVID and inverse apical and basolateral protein targeting, tethers endosomal vesicles along actin filaments and stabilizes them at the apical pole by interacting with the apical determinant Crumbs (Pocha et al., 2011). By also interacting with the small GTPase Rab11a, MYO5B controls apical trafficking and activation of Cdc42 and ezrin (Bryant et al., 2014; Dhekne et al., 2014; Roland et al., 2011). In enterocytes from individuals with MVID and in Myo5b-mutant CaCo-2 cells, Rab11a-enriched recycling endosomes no longer reach the apical pole, which likely explains the absence of apical determinants at the apical pole (Dhekne et al., 2014; Knowles et al., 2014) (Table 1). Efficient apical protein targeting requires not only trafficking and docking to the apical pole, but also fusion of the vesicles to the plasma membrane. This is achieved by interaction between soluble N-ethylmaleimide-sensitive factor attachment receptors (SNAREs) on both the vesicle (v-SNARE) and the targeted membrane (t-SNARE). In mammals, STX3 is a t-SNARE that is enriched at the apical pole (Delgrossi et al., 1997) and interacts with the v-SNARE SLP4A (also known as SYTL4) via STXBP2. MVID caused by variants in *STX3* or *STXBP2* might thus result from alterations in the apical membrane fusion machinery (Fig. 2). Whereas the molecular factors causing apical protein mislocalization are now beginning to be understood, why some of them are redirected to the basal compartment, thus leading to partial apicobasal polarity inversion, is unknown. A putative explanation is that because apical recycling endosomes are prevented from binding and fusing to the apical membrane, they instead interact with the basolateral STX4, which is highly similar to STX3 (ter Beest et al., 2005) and exclusively found in the basolateral compartments (Low et al., 1996) (Fig. 2).

Another scenario explaining partial epithelial polarity inversion involves the perturbation of cadherin-based AJ. E-cadherin engagement is an early event in epithelial cell polarization (Wang et al., 1990a). In renal epithelia of individuals with PKD, variants in the polycystins PC1 and PC2 (also known as PKD2) are thought to drive apicobasal polarity inversion by impairing E-cadherin sorting to the epithelial zonula adherens, a cell–cell junction belt ensuring mechanical anchoring of neighboring cells (Charron et al., 2000). By interacting with E-cadherin (Huan and van Adelsberg, 1999), mutant PC1 might sequester E-cadherin in the cytoplasm or destabilize it from cell–cell contacts (Fig. 2). The mislocalization of the exocyst complex proteins Sec6 and Sec8 in cells derived from individuals with PKD affects basolateral membrane trafficking and could also be responsible for failure of E-cadherin to reach the membrane (Charron et al., 2000). These data reveal that PKD-associated mutations weaken cell–cell adhesions and sustain polarity inversion. However, why AJ alteration in PKD epithelia leads to partially inverted polarity and not complete loss of the polarity axis is still unclear.

Similarly, the local inversion of polarity at bacteria–host cell contacts upon *N. meningitidis* infection involves the mistrafficking of AJ proteins to the apical pole (Coureuil et al., 2009). In response to infection, cells activate Cdc42, which recruits p120 catenin (p120ctn; also known as δ-catenin 1 or CTNND1), potentially via their shared interactor N-WASP (also known as WASL) (Rajput et al., 2013; Rohatgi et al., 2000). p120ctn repositioning at the apical pole causes relocalization of the remaining AJ proteins, leading to weakening of cell–cell junctions and increased bacterial invasion through the endothelial barrier (Coureuil et al., 2009). Here, a pathogen-driven interaction of a basolateral protein (p120ctn) with an apical determinant (Cdc42) is sufficient to drive local inversion of membrane polarity.

Altogether the scenarios presented above provide partial explanations of how mutations in genes affecting membrane trafficking, cell–cell junction remodeling and endosomal recycling might lead to inverted apicobasal polarity. Additional studies are required to better understand why in some cases polarity inversion only affects a subset of molecular determinants despite global alterations in membrane trafficking. The requirement for genetically modified animal models or samples derived from individuals with diseases for such studies has been a practical challenge for research into determinants of polarity orientation in whole epithelia compared to studies using *in vitro* cell cluster assays. Recent improvement and wider use of 3D human organoid culture will hopefully help fill this knowledge gap (Tran et al., 2022).

## Concluding remarks

Transient reversal of apicobasal polarity serves vital roles in development processes from embryo implantation to immune surveillance. Furthermore, scenarios such as host–pathogen interactions demonstrate how polarity inversion can be both a defense mechanism and a mechanism driving vulnerability to infection. Beyond these physiological roles, inverted polarity is a distinct feature of invasive cancer progression and numerous monogenic diseases. The importance of polarity inversion in the pathophysiology and molecular origin of these diseases is increasingly being demonstrated. A better understanding of the integrated pathways controlling this process is still needed in order to develop methods to restore normal polarity or artificially induce polarity inversion on demand to influence drug sensitivity, immune detection and modes of cell migration, or to improve fertility. By gathering observations made in subtypes of cancers and genetic diseases, we have provided a molecular framework to explain the development of inverted apicobasal polarity centered on the cellular response to polarizing ECM cues, actomyosin contractility and alterations in membrane trafficking. Because most of these molecular mechanisms have been uncovered from *in vitro* studies on cellular spheroids in stereotypical 3D matrices, more complex biomimetic systems involving co-culture with stromal cells are now required to further narrow down key molecular targets. Finally, recent advances in manipulation of the orientation of cell polarity (Watson et al., 2023) will greatly help bridge the knowledge gap required for therapeutic intervention in diseases featuring inverted polarity.
